# Super-Spreaders or Victims of Circumstance? Childhood in Canadian Media Reporting of the COVID-19 Pandemic: A Critical Content Analysis

**DOI:** 10.3390/healthcare10010156

**Published:** 2022-01-14

**Authors:** Sarah Ciotti, Shannon A. Moore, Maureen Connolly, Trent Newmeyer

**Affiliations:** Department of Child & Youth Studies, Brock University, 1812 Sir Isaac Brock Way, St. Catharines, ON L2S 3A, Canada; sc17kx@brocku.ca (S.C.); smoore@brocku.ca (S.A.M.); mconnolly@brocku.ca (M.C.)

**Keywords:** COVID-19, childhood, child health, media content analysis

## Abstract

This qualitative research study, a critical content analysis, explores Canadian media reporting of childhood in Canada during the COVID-19 global pandemic. Popular media plays an important role in representing and perpetuating the dominant social discourse in highly literate societies. In Canadian media, the effects of the pandemic on children and adolescents’ health and wellbeing are overshadowed by discussions of the potential risk they pose to adults. The results of this empirical research highlight how young people in Canada have been uniquely impacted by the COVID-19 global pandemic. Two dominant narratives emerged from the data: children were presented “as a risk” to vulnerable persons and older adults and “at risk” of adverse health outcomes from contracting COVID-19 and from pandemic lockdown restrictions. This reflects how childhood was constructed in Canadian society during the pandemic, particularly how children’s experiences are described in relation to adults. Throughout the pandemic, media reports emphasized the role of young people’s compliance with public health measures to prevent the spread of COVID-19 and save the lives of older persons.

## 1. Introduction

Popular media shapes public perception and awareness of social issues and groups. This empirical research explores the social construction of childhood in Canadian media reporting of the COVID-19 global pandemic. COVID-19 is a severe respiratory illness caused by a highly transmittable viral infection [1,2]. Those at the highest risk of serious complications are older adults, individuals with underlying health conditions, and those with compromised immune systems. The World Health Organization (WHO) categorizes children and youths as at risk of contracting COVID-19 as frequently as other age groups, but less likely to develop more severe forms of the disease [3].

Building upon existing global academic literature [1,2,4,5,6,7,8,9,10,11], this paper explores childhood in Canada during the 2019 coronavirus (COVID-19) global pandemic. COVID-19 led to a significant global health crisis [12]. The long-term health and social effects of the pandemic remain unknown. Children present with milder cases of COVID-19, and more severe symptoms and death are extremely rare [7]. For instance, “patients aged < 18 years only account for 2% of severely affected patients” [10]; however, emergent research suggests that COVID-19 may be linked to a ‘multisystem inflammatory state’ in children [8]. In 2020, the WHO recommended that young people avoid contact with those who are at a high risk of severe disease given that they are known vectors of the disease and can be asymptomatic. In examining the social construction of childhood in Canadian media reporting of the COVID-19 pandemic, we were guided by a central question: what is the impact of the COVID-19 global pandemic on children in Canada? By analyzing media reports of childhood during the first wave of the pandemic, we gain a deeper understanding of its impact on children and adolescents.

Our theoretical framework combines the social determinants of health as outlined by the World Health Organization (WHO) and the social construction of childhood [13,14,15]. According to the WHO (2008), “the social determinants of health (SDH) are the non-medical factors that influence health outcomes. They are the conditions in which people are born, grow, work, live, and age, and the wider set of forces and systems shaping the conditions of daily life” [3]. In other words, social conditions account for the differences in individual and public health. The social determinants of health are particularly significant in understanding children’s health: pediatric guidelines have recognized the role of adverse social determinants of health for over 25 years [16]. COVID-19 caused a significant public health issue in Canada (and globally), but the impact of the pandemic on individuals and communities must be understood in a socio-cultural context.

## 2. Materials and Methods

This qualitative cross-sectional [17] study explored online Canadian media reports of childhood in the COVID-19 global pandemic by utilizing inductive, critical content analysis [18] and thematic analysis [19,20] as methods. A critical content analysis explores issues of “power in social practices by understanding, uncovering and transforming conditions of inequality and locating sites of resistance and change” [18]. A thematic analysis “is a method for systematically identifying, organizing, and offering insight into patterns and meaning (themes) across a data set” [20] to address a research question. Thematic analysis is a flexible method, compatible with critical content analysis as it allows researchers to “make active choices about the particular form of analysis they are engaged in” [19]. “Cross-sectional studies are carried out in one point or over a short period of time... They are usually conducted to estimate the prevalence of the outcome of interest for a given population, commonly for the purposes of public health” [17]. A qualitative approach allows for a deeper level of analysis of media content, including identifying dominant themes as well as the context and tone of the media coverage. 

### 2.1. Sampling

The data set included Canadian news media reports accessible via online databases. We searched the popular online databases Google and Facebook using the following search terms: “COVID-19”, “childhood” and “Canada”. We were particularly interested in reports that were widely disseminated to the Canadian public and available online. Google and Facebook were specifically selected given that they are free and publicly accessible. Worth noting, all major Canadian news sources and governmental agencies (at the provincial, territorial, and federal levels) are accessible through these platforms. For example, the Government of Canada, Health Canada and all Canadian provinces and territories have Facebook pages and official websites that are accessible via Google search. During the first wave of the pandemic, access to print media and retail outlets was limited. Canadians were encouraged to stay home and reduce non-essential trips in the community; thus, online platforms became important instruments for information sharing by citizens, researchers, academics, public health organizations and governments globally. 

**Figure 1:**
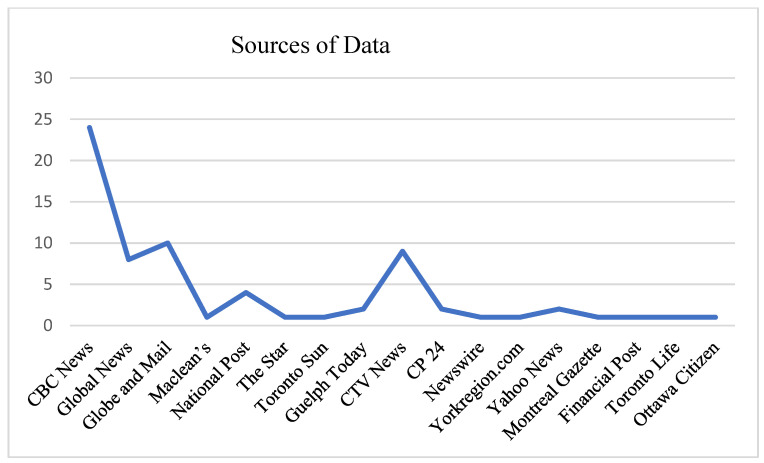
Sources of Data.

Non-Canadian media, anecdotal websites, and websites that did not contain COVID-19 related information were excluded. This data set was inclusive of 70 online news media reports on COVID-19 and childhood from 17 different national and local news media outlets (both publicly and privately funded), ranging from large well-established publications to small community-based publications. We acknowledge the data sources capture a specific period through which the data was collected: specifically, the dates of publication fell within the first wave of the pandemic (between 12 March 2020 and 11 August 2020). In Figure 1, the sources of data are identified by the name of each publication and the number of articles reviewed.

### 2.2. Analysis

We analyzed Canadian media reports of childhood and COVID-19 from the first wave of the pandemic. It is important to understand how the roles of children are socially constructed in a society. Deconstructing representations of childhood in Canadian media reporting of COVID-19 provides a deeper understanding of the wider impact of this public health crisis in Canada and contextualizes the overall impact of the pandemic on Canadians. Given the pandemic was a worldwide crisis, the results from this study are applicable to the social construction of childhood globally. 

Content analysis is relevant in mass communication research [21] because “media content gives us a blueprint of the cultural codes in societies because all facets of the mass media are created by humans, people with conscious and unconscious perceptions” [22]. The media is a representation of the dominant social discourse and socio-political ideology. Thus, misinformation flow and correction must be understood in the context of the broader culture [23]. Furthermore, “in highly literate societies, written texts provide particularly telling windows into social worlds” [24]. In Canada, the print media serves an important function in disseminating knowledge, thus, it is essential to explore how complex social issues, such as health issues, are broadly understood. During the first wave of the COVID-19 pandemic, mandated public health strategies were implemented by the government to stop the spread of the disease within communities.

## 3. Results

The results of this study point to two central themes that emerged through the data analysis: the role that children and adolescents play in the community spread of COVID-19 (children “as a risk”) and the impact of the pandemic on children’s physical and mental health (children “at risk”). Children are largely understood as vectors of the disease with a significantly lower risk of serious illness or death. Although adults are at significantly higher risk of serious complications from the disease, children and adolescents were acutely impacted by the pandemic: if not directly from contracting the disease, then from the resulting social and structural changes. As Maclean’s reported on 11 June 2020, “the pandemic may have mostly spared kids, but there are few groups whose experience of the world is changing so dramatically as a result” [25]. To this point, on 17 June 2020, CTV News reported that “some of those consequences include decreased vaccination coverage, delayed diagnosis and care for medical conditions unrelated to COVID-19, impacts on children’s behaviour and mental health, and exposure to child abuse, violence, and neglect” [26]. Children and adolescents faced both direct and indirect consequences of the pandemic: their mobility and community access were restricted to stop the spread of the disease and therefore they had less access to support and resources outside of their homes. For some, the pandemic presented an increased risk of family violence, abuse, neglect, and consequently adverse mental and physical health outcomes.

### 3.1. Theme 1: Children “as a Risk”

Early in the pandemic, the Canadian media paid significant attention to the role of children as vectors of COVID-19—this has persisted throughout each wave emphasizing both the novelty of the disease and the need for further research on its impact on children. Many media reports emphasized the fact that children appear to be less likely to show symptoms and those who are symptomatic have less severe symptoms. The narrative of children as vectors evolved in real-time over the course of the pandemic. For example, CBC News (2020) reported on 25 May 2020: “kids are not only less likely to be infected...but they are also less likely to transmit it. Adults are more likely to transmit the virus to kids than the other way around” and “there has been confusion over the evolving science on the amount of asymptomatic transmission since the start of the pandemic” [27]. Later, a CBC News report on 9 June 2020 asserted that “children are going to be an important part of the whole story over time of COVID-19” [28]. By August 2020, as schools prepared to re-open in September, some media reports began to suggest that children may increase the community spread of COVID-19 if they returned to the classroom. This marked the beginning of the discourse regarding children as “super spreaders” of COVID-19, which seemed to originate in the United States, as the country was grappling with re-opening schools to in-person learning whilst managing the continued increases in community spread of the disease. 

The social discourse about children “as a risk” included discussions of the importance of mass vaccinations for COVID-19 globally. Vaccines play a critical role in stopping the spread of COVID-19. Given the novelty of the virus, there were no vaccines developed before the pandemic was declared. However, during the first wave of the pandemic, the global scientific community and pharmaceutical companies began to research and develop COVID-19 vaccines. Globally, governments contributed substantial financial investments for the acquisitions of reliable vaccines. By May 2020, there were at least 6 vaccine candidates in clinical trials globally [5], and by July 2020 “there (were) almost two dozen vaccines in clinical trials around the world and at least 140 more in earlier stages of development, but most experts predict it will be well into 2021 before the first vaccines could be ready for wide use” [29]. In December 2020, Health Canada approved two vaccines for COVID-19 and began disseminating them to higher risk and vulnerable populations, including elderly patients in long-term care, older adults living in the community, adults in Indigenous communities and adults with underlying health conditions. Although COVID-19 vaccine approvals in children and adolescents began on 9 December 2020 (when Health Canada approved the Pfizer-BioNTech Comirnaty COVID-19 vaccine for young people of 16 years of age and older), it was not until 5 May 2021 when Health Canada approved the Pfizer-BioNTech Comirnaty COVID-19 vaccine in individuals from 12 to 15 years of age, and not until 19 November 2021 that Health Canada approved a lower dose of the Pfizer-BioNTech Comirnaty COVID-19 vaccine for use in children aged from 5 to 11 years [30]. Importantly, the growing anti-vaccine movement and parental refusal to vaccinate their children [31,32] presents a significant barrier to the mass vaccination of children and adolescents. The pandemic has highlighted how online and social media platforms have fueled the anti-vaccine movement by spreading propaganda and misinformation through these outlets [33,34]. Given these challenges, distancing measures may continue to play a critical role in public health. Countries that adopted physical distancing measures were better able to mitigate community transmission [11]. The Government of Canada (2020) implemented distancing measures aimed at stopping the community spread of COVID-19. Media reporting emphasized Canadians’ civic duty to comply with public health guidelines to stop the community’s spread of the virus.

### 3.2. Theme 2: Children “at Risk”

The physical and mental health effects of COVID-19 on children and adolescents were discussed in media reports. Three sub-themes emerged from the data: the mental health effects of social isolation, “multisystem inflammatory syndrome” in children and the health consequences of decreased physical activity because of quarantine protocols. Reports of the physical health effects of COVID-19 in children emphasized the lower risk of infection and serious consequences, including a 13 June 2020 Globe and Mail report that “no Canadian under the age of 19 is known to have died of COVID-19” [35]. Nonetheless, media reports, including one from CTV News on 25 June 2020, acknowledged that “while children and teenagers appear to be less likely to be afflicted with severe COVID-19, new research is warning of the several indirect consequences the pandemic is having on their physical and mental health... From delays in seeking proper care for an illness unrelated to COVID-19 to a heightened risk of family violence, countries...the pandemic response measures have taken a substantial toll on the well-being of children around the world” [26].

In other words, although the disease itself presents less of a risk of serious consequences for young people, pandemic measures impacted the daily lives of children and adolescents in Canada and globally. 

The impact of the pandemic on children’s mental health was largely articulated in terms of the impact of school closures and increased pandemic-induced stressors. For example, on 15 June 2020 and 16 June 2020, CTV News published two separate reports that revealed that children were regressing in their development due to an increase in stress from the pandemic [36,37]. On 17 June 2020, CBC News reported, “Kids Help Phone has seen a surge in calls and texts from young people needing someone to talk to during the COVID-19 pandemic” [38]. Then, on 7 July 2020, the Ottawa Citizen reported, “when it comes to mental health treatments... the pandemic has contributed to a surge in demand for services...the impact of COVID-19 on children has been underestimated... (in particular) the impact of isolation on mental wellness and development” [39]. In several articles, schools were identified as important institutions for children’s mental health support. For example, Global News reported on 19 May 2020 that Ontario’s Education Minister, Stephen Lecce, “acknowledged that the pandemic has been ‘tough on children.’ He nodded to the province’s CAD 12 million investment in mental health amid COVID-19 as part of what can assist educators and kids” [40]. CBC News reported on 5 June 2020, that “the idea is for teachers to touch base with students, from reviewing lessons that may have been challenging via remote learning to checking in on students’ mental health amid the pandemic” [41] and on 10 June 2020, they reported “Physicians who work with families and children are concerned about students’ mental health, the CPS also said, and worry about how they’ll fare in the new school year” [42]. The social discourse regarding children’s mental health brought to light the challenges young people faced as a specific consequence of the pandemic. 

As mentioned, media reports drew attention to “multisystem inflammatory syndrome”, a distinctive COVID-19 complication in children. On 13 May 2020, CP24 News reported that “COVID-19 will now include multisystem inflammatory vasculitis, which may appear in children...the connection between this inflammatory illness and COVID-19 is not confirmed at this time...the majority of COVID-19 infections in children are mild and do not require hospitalization” [43]. Then, on 18 May 2020, CTV News reported, “the return (to school) has put some children in new danger of infection...the idea of children being silent ‘super-spreaders’ has been largely debunked...last week France recorded its first death of a child linked to Kawasaki disease, a mysterious inflammatory syndrome that some doctors say could be triggered by COVID-19” [44]. A week later, on 25 May 2020, CBC News reported, “hundreds of cases of the Kawasaki-like disease have been detected around the world...young children seem to be at the least risk of contracting COVID-19, and when they do, they seem to suffer less severely... Kids are not only less likely to be infected...but they are also less likely to transmit it. Adults are more likely to transmit the virus to kids than the other way around” [27]. Thus, pointing to the construction of children’s pandemic in relation to adulthood.

The impact of the pandemic on children’s physical health was described in the context of lockdown restrictions. On 17 June 2020, a CP24 News report argued that “before the pandemic, Canadian children were barely getting a passing grade for overall physical activity and sedentary behaviours...Restrictions from the COVID-19 crisis have made things even worse...children and youth in Canada are not as active as they should be and have too much screen time...only 39 percent of children (aged five to 11) and youths (12 to 17) met the national physical activity guidelines of 60 min of moderate to vigorous physical activity per day” [45]. Then, on 25 June 2020, CTV News reported that “less social interaction and a lack of structured routines can result in increased screen time, reduced physical activity, and higher rates of depression and anxiety among children” [46]. Lockdown restrictions during the first wave of the pandemic restricted children’s access to extra-curricular and school-related physical activities; moving education online increased screen time and sedentariness for children and adolescents. The pandemic lockdowns contributed to decreased physical activity in children and adolescents, which is important for their overall health and wellbeing (both physically and mentally).

## 4. Discussion

The purpose of this study was to gain an understanding of the impact of the COVID-19 global pandemic on children and adolescents in Canada. The COVID-19 pandemic caused significant concern globally and nationally. The social discourse regarding COVID-19 evolved over the course of several months. Populations and communities rely on the mass media to disseminate information related to COVID-19, including how to prevent and treat the disease. Given the novelty and the rapid and vast spread of the disease, researchers, health care providers, governments and the public initially struggled to understand and prevent community spread. The media played a significant role in disseminating COVID-19 information to the public. The management of this crisis, particularly, the propagation of information, draws attention to the challenges presented in distributing rapidly evolving information on a global, national, and international level. As knowledge increased and information evolved, Canadians were required to be flexible and adaptable. Pandemic responses drew criticism, sparked controversy, and gave rise to conspiracy theories and resistance directed at the government and medical community. For example, initially, Dr. Theresa Tam, The Public Health Agency of Canada’s Chief Medical Officer, publicly cautioned against the safety and use of cloth masks. As the Toronto Sun reported on 30 July 2020, Dr. Tam advised that masks were pointless and risky on 29 January 2020; it was not until 22 April 2020 when the Public Health Agency of Canada recommended Canadians wear non-surgical masks in public to protect themselves and others from COVID-19 [47]. Arguably, this sudden change may have been a factor in the rise of ‘anti-maskers’ [48] who vocalized their displeasure with mandatory mask by-laws and argued their rights were being infringed upon, mainly attributed to the spread of COVID-19 conspiracy theories online. Mask debates expose the critical role that social media plays in shaping and influencing social discourse as well as spreading information, including inaccurate and potentially dangerous rhetoric. Media consumers are therefore tasked with fact-checking the sources of the information they consume online. This can be difficult given that some alternative media sources invest significant resources in attempting to appear legitimate. Furthermore, given that information is shared so rapidly, it is arguably very difficult to vet all information consumed on social media channels.

For some families, the COVID-19 pandemic led to increased daily stressors, including family conflict, employment and economic stress [49,50,51,52]. Lockdowns have been associated with increases in intimate partner violence [53,54,55] and exposure to family violence is harmful to the health and development of children and youth [56]. Additionally, socioeconomic inequity and poverty adversely impact health and wellbeing [57,58]. The pandemic highlighted the economic insecurity faced by Canadian families, particularly low-income families, and families with high levels of household debt [59]. Distancing measures resulted in job losses and layoffs due to forced business closures of non-essential services. Families with children in school and daycare immediately lost childcare, creating employment barriers. Single-parent families with young children were significantly disadvantaged by this loss of childcare. Childcare responsibilities disproportionately fell on working mothers who were tasked with juggling work from home employment expectations and childcare responsibilities [49]. Family stress is associated with adverse childhood experiences and negative health outcomes in childhood and adulthood [57], which points to the unique impact of the COVID-19 pandemic on children and youths in Canada.

### 4.1. Recommendations

The findings from this study point to the need for further research examining the long-term impact of the COVID-19 pandemic on children and adolescents. Researchers can learn a great deal from children and adolescents regarding the effects of the pandemic. As child studies scholars have long argued, children’s participation in research is essential to accurately represent their embodied experiences in their own voices [13,60]. Childhood is not universal and children’s experiences are not homogeneous, thus, childhood must be understood in terms of the broader social and cultural context [61]. Research evaluating the impact of the pandemic on diverse communities in Canada and research that focuses on those individuals who have been traditionally underrepresented, marginalized and excluded from dominant social discourse should contribute to current academic knowledge concerning the impact of the pandemic on Canadians. This includes young people from racialized communities, including Black and Indigenous youth, as well as young people with disabilities, those from lower socio-economic status families, children and youths in foster care, homeless youths, and those who identify with the LGBTQIA+ community. The data analyzed in this research suggest that children and adolescents have been uniquely impacted by the pandemic and the extent of this impact remains largely unknown. To fully grasp the impact in Canada, it is important to explore the impact on young people from diverse communities across the country.

We recommend further studies exploring how mass media and, in particular, social media has influenced COVID-19 vaccine hesitancy. We assert that the COVID-19 global pandemic underscores the role of accessible, free and unbiased media. The advent of social media has shifted how the general public access news; increasingly, people are accessing news media content via social media platforms online [23]. This is potentially problematic, as social media content blurs the line between personal opinion and objective, fact-checked news [62]. A prominent example, the US presidential election, underscored the extent to which social media content has perpetuated the rise of ‘fake news’ discourses [63]. Social media amplifies misinformation and presents a challenge for researchers attempting to disseminate scientific data [33]. In the early stages of the COVID-19 epidemic, the media played a critical role in increasing community awareness of the public health threat as well as efforts implemented to control the spread. Citizens, health professionals, scientists and governments shared information via the media and social media platforms. The increased accessibility that social media platforms offer can be an asset—knowledge is disseminated more rapidly, which, in turn, increases public awareness and civic engagement. These factors contributed to citizens and communities abiding by distancing measures that helped to contain the spread of this infectious disease. However, as the pandemic has unfolded, social media has been a significant driver of pandemic-related information and misinformation [34], presenting challenges for governments, public health officials, researchers and the general public. We argue this will continue to warrant further academic investigation.

### 4.2. Limitations

Media reports are open to interpretation and therefore this analysis is subjective and informed by the philosophical assumptions of the authors. Although attempts were made to access all Canadian media content on childhood and COVID-19, the data were limited to information gathered via the specific search engines and search terms used. Given the regional health guidelines for social distancing and quarantine protocols due to the COVID-19 pandemic, this paper is limited to content from media available online. The disease remains a significant global concern and this study is inclusive of the information available at the time. We acknowledge that knowledge and research on COVID-19 will continue to progress following the completion of this research study.

A significant limitation of this study is the small sample size, given this, the authors are in no way suggesting that the results of this study are generalizable. The data gathered represent a narrow window of time (during the first wave of the pandemic); therefore, results can be characterized as a snapshot of the dominant social discourse at the time. Published media reports following the first wave of the pandemic would contribute insight and build upon prior existing knowledge about the impact of the pandemic on children and adolescents in Canada. As a diverse, multicultural country, the pandemic has highlighted existing inequities for racialized communities [64]. Finally, as mentioned, childhood is not a universal social construct and therefore the findings from this study are not generalizable for the entire population of individuals under the age of 18 globally. 

While the data analyzed in this study were focused on the first wave of the pandemic, each wave placed restrictions on children and youths, including their mobility and access to their friends, extended families, and community. With each lockdown, young people in Canada were amongst the first groups to be impacted, from closures of schools and online learning to restrictions on social gatherings and closures of community programs. Although this group is at lower risk of complications and death from the disease, children and adolescents continued to be disproportionally impacted by public health guidelines that meant to stop the community spread whilst having little input and opportunities for civic engagement regarding decisions impacting their health and wellbeing. Thus, this further highlights the importance of research that represents children and adolescent experiences of the pandemic in their voices.

## 5. Conclusions

Canadian media reporting of COVID-19 and children encapsulates the impact of the COVID-19 pandemic on the country’s socio-economic, political, and healthcare systems more broadly. Emerging from the data are constructions of childhood and adolescence bounded by other social systems, including family, education, healthcare, political and economic systems. The overall impact of the COVID-19 pandemic is unique for young people with variable health effects in terms of their mental and physical health. The findings from this study demonstrate how mass media reflects socio-cultural discourse in a society. In the Canadian context, childhood is contextualized by adulthood and constructed through an adult-centric lens. Specifically, social discourse pertaining to childhood was framed in terms of ‘risk’, thus missing other aspects of children’s experiences in their own voices. To accurately understand issues related to children and adolescent health, researchers must engage children as active participants in health research. Knowledge regarding the COVID-19 global pandemic continues to evolve, thus we cannot speculate with any certainty the long-term effects on children, adolescents, and adults. The consequences of the COVID-19 global pandemic are likely to inform social and health policy in Canada for years to come. We suggest further research exploring the impact of the pandemic on children and adolescents.

## Data Availability

Publicly available datasets (news media web pages) were analyzed in this study. The data presented in this study are available on request from the corresponding author.
